# Innovative forest products in the circular bioeconomy

**DOI:** 10.12688/openreseurope.14413.2

**Published:** 2022-06-16

**Authors:** Mariana Hassegawa, Anna Karlberg, Magnus Hertzberg, Pieter Johannes Verkerk

**Affiliations:** 1 Bioeconomy, European Forest Institute, Joensuu, 80100, Finland; 2 SweTree Technologies, Umeå, 907 36, Sweden; 3 Stora Enso, Falun, SE-791 80, Sweden

**Keywords:** bioeconomy, wood, forest products, innovation, circularity, sustainability

## Abstract

**Background: **The forest-based industry has been moving towards the manufacture of bio-based products in response to the increasing concern by consumers and governments regarding the use of non-renewable materials and the generation of residues. Various innovative technologies geared towards reducing the environmental footprint of products and processes are currently being developed and applied in the forest-based industry. This study presents some innovative wood-based products that are about to enter the market or that are already being commercialized but have the potential to expand in market size.

**Methods: **We collected data from interviews and a survey with organisations working with product development and manufacturing, and from the literature.

**Results: **Many innovative products that are already produced at an industrial scale, such as cross-laminated timber, wood-based composites, and lyocell, can still increase their market share in the coming years. Some of the up-and-coming products with high potential to substitute fossil-based materials and will likely enter the market in the near future are wood foam, lignin-based adhesives, glycols, bioplastics, and textile fibres. Our study indicates that, although biomass demand is expected to increase, stakeholders do not consider future supply a limiting factor.

**Conclusions:** The ease of market introduction of innovative products relies heavily on the products’ ability to take advantage of existing value chains. Overall, many of the reviewed products have the advantage of being ‘drop-in’. This is because products that require adjustments to production lines are less likely to get into the market without strong external drivers that push for bio-based alternatives. According to stakeholders, the economic viability and the market expansion of these products could be encouraged to a certain extent by EU policies, and certain barriers could be alleviated by reducing bureaucracy, increasing the support for pilot-scale to full-scale production, and increasing subsidies for bio-based alternatives.

## Introduction

Manufacturing value-added bio-based products has become increasingly important to the forest industry in response to the growing awareness on environmental problems related to emissions of greenhouse gases (GHG) and the generation of waste and pollution ( Hurmekoski *et al.,* 2018; Lettner *et al.,* 2018). New technologies and products that reduce carbon footprints and tackle pollution and waste production are being developed ( Sathre & O’Connor, 2010). Emphasis is also being put on products that are sustainable, biodegradable and that can be recycled. This shift is very much in line with the circular bioeconomy, the main elements of which are the use of biological resources, their substitution for fossil sources to produce energy and other manufactured goods, the use of biotechnology, all taking into consideration the end-of-life stage of products, thus steering away from the traditional linear economic models that assume infinite supply of resources ( Reichel *et al.,* 2016).

Bio-based products are wholly or partly derived from primary biomass or from the derivatives and by-products from a biomass transformation process. Because of variation in the carbon content from biomass, the products are frequently characterized by their bio-based content or bio-based carbon content ( Willemsee & van der Zee, 2018). These products should also be sustainable, regarding the sourcing of feedstock, product design, the production process, waste, residue treatment, among others. Other aspects to be considered are the use of energy, water and other resources and the emission of pollutants during processing or manufacturing a product, to ensure that the bio-based products will have low carbon and water footprints ( Weiss *et al.*, 2012). Bio-based products are often an improvement from older technologies or from fossil-based products ( Mekonnen *et al.*, 2013; Winandy & Morrell, 2017), in terms of reduced emissions of GHG and other pollutants, and waste, among others.

In addition to the need to reduce GHG emissions associated with products, one important concern in the design and manufacture of products relates to the use of plastics that release microplastics during regular use and after being discarded ( World Economic Forum, 2016). Microplastics, as is the case for synthetic materials such as polyesters, are an important cause of water pollution ( Ellen MacArthur Foundation, 2017). Because of these concerns, the European Union (EU) has banned the production and consumption of single-use plastics ( European Parliament and Council of the European Union, 2019). Since this EU directive applies to all plastics produced with chemically modified polymers, whether from natural or synthetic sources, it would also have implications on bioplastics.

In addition to the need to reduce GHG emissions associated with products, one important concern in the design and manufacture of products relates to the use of plastics that release microplastics during regular use and after being discarded ( World Economic Forum, 2016). Microplastics, as is the case for synthetic materials such as polyesters, are an important cause of water pollution ( Ellen MacArthur Foundation, 2017). Because of these concerns, the European Union (EU) has banned the production and consumption of single-use plastics ( European Parliament and Council of the European Union, 2019).Since this EU directive applies to all plastics produced with chemically modified polymers, whether from natural or synthetic sources, it would also have implications on bioplastics.

Transient consumer preferences and policy redevelopments are pushing industries to develop technologies, processes and materials that are more sustainable and less harmful for the environment. One way to at least partially achieve this is by using renewable feedstocks to produce functionally equivalent materials that can displace resource-intensive products. For instance, fossil-based plastics and composites used in packaging are being substituted by bioplastics ( Philp *et al.,* 2013) and cellulose-based foam ( Hjelt *et al.,* 2021). Cement, bricks and steel can be partially substituted by wood structural elements in construction of buildings ( Antikainen *et al.,* 2017; Churkina *et al.,* 2020; D’Amico *et al.,* 2021; Green & Taggart, 2017). The textile industry is looking into producing and using textile fibres that are less resource-intensive, cause less pollution and can be recycled at the end of the product lifecycle ( Kataja & Kääriäinen, 2018).

There is a wide range of products that can be manufactured from woody biomass (e.g., Verkerk *et al.*, 2022). However, some of these products and technologies are either in early stages of development or are, as at time of writing, deemed technically or economically unfeasible. Previous studies have tried to identify new and emerging bio-based products, but were not specific to the forest sector ( COWI *et al.,* 2019; University of Bologna & Fraunhofer ISI, 2018), or they focused on particular product categories such as chemicals ( Lettner *et al.,* 2018; Spekreijse *et al.,* 2019). Hurmekoski *et al.* (2018) looked at new products manufactured by the forest sector, but focused on Finland, Sweden, the United States and Canada.

This study aimed to identify innovative forest products in the EU, with market potential, that could possibly contribute to climate change mitigation through substitution. More specifically, we aimed to answer the following research questions:

- What are the main forest products that could be economically produced in the EU from forest-based lignocellulosic biomass in the near to midterm future?
- What fossil-based chemicals or materials could the innovative forest products substitute?
- What are the feedstock requirements for biomass quality and quantity?
- To which extent are these forest products compatible with existing value chains?

## Methods

### Scope of the analysis

In a circular bioeconomy, forest biomass can be used as feedstock for materials and energy, which can substitute for more emissions-intensive, non-renewable materials. In this study, we focused on products from the forest-based sector, that were manufactured either from wood (e.g., solid wood, wood chips, fibres) or from industrial side streams (e.g., black liquor, sawmill residues). Despite the fact that forest products can substitute fossil-based products in a large number of applications, some have a higher chance of entering the market or increasing in market share than others. In addition, a multitude of products could be classified as “innovative forest products”. To help narrow down the number of options of products to be reviewed, we defined the following criteria:

- the feedstock used to manufacture the product should be derived from wood or from by-products obtained during the industrial processing of wood;
- the products should cover a range of product types from five categories, namely: construction materials, textiles, chemicals, bioplastics and composites; and
- the products should have a Technology Readiness Level (TRL) between 5 and 9.

Because of the overlap between some product categories, we considered that “construction materials” would encompass elements used for structural (e.g., engineered wood products) and non-structural purposes (e.g., insulation materials) in buildings. We grouped products such as fibreboards and laminated veneer lumber, which are frequently denominated “composite materials” for being composed of wood elements bonded with adhesives ( Stark & Cai, 2021), in the category “construction materials” due to their applications. The category “composites” would include all products where small wood elements (i.e., wood chips, particles, fibres, and woodmeal) are bonded with a binding agent to form products that are purposes other than building construction. “Textiles” would encompass staple fibres and filaments, as well as threads, yarns and fabrics made of wood-based fibres, typically used for clothing and household items. Products that contained textiles combined with other materials, such as cement, foam, resins, etc., were not included in this category for being considered composite materials. The category “chemicals” focused on chemical compounds produced from biomass or through a bioprocessing route ( Philp *et al.*, 2013). Cellulose, which is technically a chemical compound, was not included in this category as it is a fundamental wood component. However, chemicals produced from cellulose (e.g., platform chemicals, were considered potential products in our study ( Geboers *et al.*, 2011; Nitzsche *et al.*, 2021; Takkellapati *et al.*, 2018). Finally, “bioplastics” comprised products and materials made of bio-based polymers from woody biomass and its derivatives that had similar properties to conventional plastics ( Vert *et al.*, 2012).

We used the TRL proposed by the European Commission (2014) as an indicator of the stage of development of the product or technology. Based on this classification, we estimated that products with low TRL (between 1 and 4) would take more than 20 years to become commercially feasible, if they ever became technologically and financially viable ( Hurmekoski *et al.,* 2018). Medium to high TRLs (>4) were estimated to have potential of entering the market in the next 20 years.

Considering these criteria for defining innovative forest products, a first list of potential products was created using scientific and grey literature, news articles, and websites of major companies and research institutions. This preliminary list was improved upon with a structured web search using keyword blocks, such as:

a) Keywords block one: “forest-based” OR "wood-based" OR "bio-based" OR "wood";
b) Keywords block two: term related to the product category (e.g., for category “construction materials”: “construction materials” OR “construction” OR “building materials” OR “building elements”).

While compiling the list of potential products, we also checked which research institutes and companies that were involved in the development or manufacture of bio-based products in the EU. The list of potential stakeholders was built by consulting the member organisations of confederations, consortia, and wood industry associations, such as Innovawood, the European Confederation of Woodworking Industries (CEI-Bois), and the Bio-based Industries Consortium (BIC), to name a few. This list of potential organisations was improved upon based on expert knowledge by including well-known companies and research institutes that were active on the development of new technologies. Once the preliminary list of potential products and stakeholders was built, we proceeded with the design of the data collection methodology, which would be composed of interviews with stakeholders, literature review and an online survey with stakeholders.

### Data sources

Information on the development and characteristics of new or emerging products and technologies is not often publicly available. Therefore, we collected qualitative information and quantitative data using a combination of data collection methodologies, including interviews, a survey, and a scientific and grey literature review. Firstly, the semi-structured interviews would allow us to obtain more detailed answers from the participants ( de Leeuw, 2008) and go deeper into discussions about products and aspects that were deemed more important to the organizations developing the products. Secondly, the online survey provided participants the flexibility to respond at their own time and pace, allow for anonymity, and reach a larger and more diverse group of participants ( Braun *et al.,* 2021). Thirdly and finally, the literature review allowed us to obtain specific information about products that were mentioned during the study. These multiple methods of data collection would help reduce the errors associated with using a single method ( Patton, 1999), by allowing us to triangulate the data from these three sources ( Tassinari *et al.,* 2021). examine the data for consistencies and discrepancies ( Barnum, 2021; Mathison, 1988), and increase the robustness of our results. A thematic analysis was performed to the qualitative data ( Bengtsson, 2016). The COREQ guidelines ( Tong *et al.,* 2007) were adopted to throughout this study. The procedure adopted in each of these methods is described in the sections that follow.

***Interviews.*** Since our study focused on assessing new and novel products, and because there is currently a myriad of forest products available on the market and under development, a direct contact with product developers through interviews was deemed the most efficient avenue for product scoping. As previously described, we compiled a preliminary list of products and organizations developing and manufacturing innovative forest products. To reduce bias towards one or few EU regions, we made sure the listed organizations were operating in several countries within the EU.

As the main objective of the interviews was to scope for new products and considering that organizations are usually involved in the development of several products, we decided that, from each product category (i.e., construction materials, textiles, chemicals, bioplastics and composites), two organizations would be selected for an interview. From a pool of 39 stakeholders, we selected 12 organizations based on the variety of products in development and the geographical location of their facilities. After establishing contact by email, 10 stakeholders agreed to participate in our interviews.

The interview questionnaire was built with the main objective to scope for innovative products being developed by the organization. We also aimed to collect qualitative data about the production process and the value chain, as well as to get insights about the current and future markets. The organizations also indicated, whenever possible, the time-to-market and TRL. The interviews were semi-structured, using the questionnaire as the basis, but opening the discussion according to the stakeholder’s interest. The interviews were conducted by two researchers, one being responsible for moderating the interview and another for giving support, especially with note taking. Moderation was done by authors M.Ha., A.K., and M.He, and support by authors M.Ha. and A.K.

At the time of data collection, M.Ha. (female) was a researcher with a PhD in Wood Science, A.K. (female) was Chief Project Officer with a PhD in Plant cell and molecular biology, and M.He. (male) was Business Area Manager with a PhD in Plant cell and molecular biology. All authors involved in the interviews had previous experience with data gathering for qualitative research. Apart from two cases, there was no prior relationship established between the researchers and the interview participants. As the main objective of the interviews was to scope for new products, we believed the potential interviewer bias in these two instances would not compromise the data quality. The interview participants were informed about the interviewers’ qualifications and the general purpose of the study both in the communications by email and at the time of the online interviews. The interviews lasted for about one hour and the notes were recorded in written format. When not moderating, authors M.Ha. and A.K. were mainly responsible for writing down participants’ answers and insights.

***Literature review.*** The list of products was built upon the preliminary list stemming from the interviews by scoping for products on websites of the manufacturing companies and research institutes, and in the literature. The selection of products to be included in the list was based on their estimated TRL (5–9), and time to enter the market (for new products) or potential to increase in market size (for novel products). We also took into consideration whether they were niche products targeting a specific market segment or a broad consumer market, and the circularity aspects of the product and production process. We focused on products that could be manufactured in the EU in the short to medium term, i.e., that could potentially enter the market or expand the market size in the next five to 10 years. Once the products were selected, we conducted a review of scientific and grey literature, as well as websites of the manufacturing companies and research institutes. The information collected during the review included – but was not limited to – quality and quantity of feedstock, general description of production process, global demand and production quantities, TRL and time-to-market (for new products), and end-of-life options.

***Survey.*** As the next step, we complemented the information collected during the interviews and literature review with an online survey. We developed the survey to collect qualitative and quantitative data on feedstock, transportation distance from feedstock source to mill, TRL and time-to-market, estimated production volume, information on substitution, among other information. The 39 organizations from the industry and research institutes that formed the pool of stakeholders in this study were contacted through email and asked to complete an electronic survey on SurveyMonkey. We collected 11 responses, which we deemed too low. To increase the number of responses, we duplicated the survey, however with a different link. The link to this separate survey was shared on social media channels from the EU BioMonitor project, as well as those of the European Forest Institute and nova-institute. By creating a separate survey, we would be able to distinguish the answers by stakeholders from the ones given by organisation representatives through social media to increase the effectiveness of screening for ambiguous or incomplete data.

### Analysis

**Figure 1.**
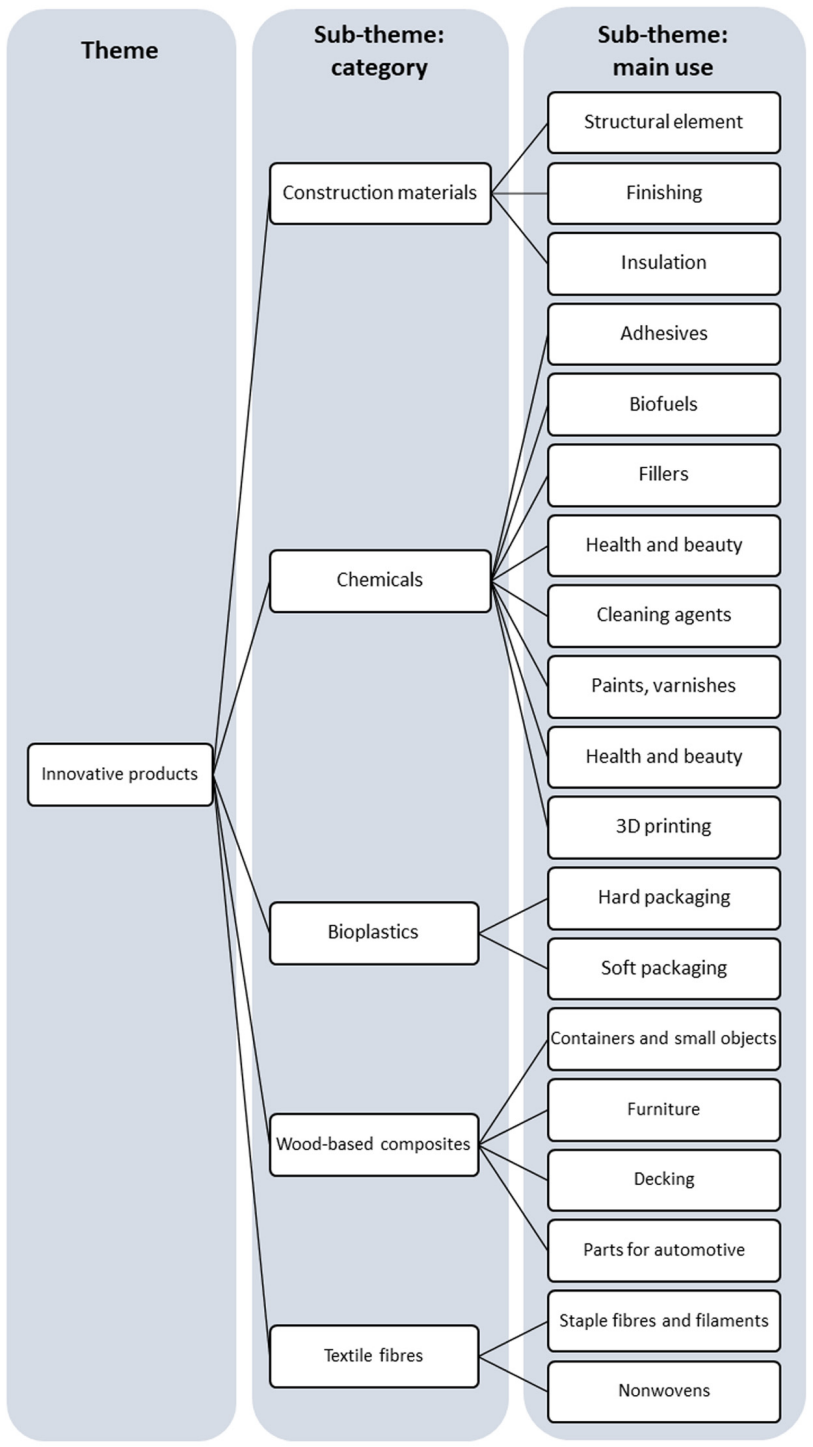
Major theme and sub-themes derived from the semi-structured interviews with stakeholders.

The information collected during the literature review on forest products and the data obtained from the research centres and industry representatives were combined to assess the potential implications of the new forest products entering the global markets. A coding system was used to categorize the qualitative data, as described by Tassinari *et al.* (2021). As the stakeholders used different terms for the same object, we harmonized the content as much as possible, making sure information was not lost in this process. Coding was performed by M.Ha. and A.K. using a hybrid coding approach, where a deductive approach (i.e., pre-established codes) was used for the sub-theme “category” and an inductive approach for the sub-theme “main uses”. A coding tree was built with the major theme and sub-themes identified in the semi-structured interviews ( Figure 1). Due to the objective of the interviews and the nature of the collected data, participant feedback was not deemed essential for the integrity of the study and thus was not performed.

We performed a stepwise backward elimination of the products mentioned by the participants, by (i) removing products that did not use feedstock from the forest sector, (ii) that had low TRL, or (iii) that were already on the market but with little or no prospects of expanding their market size. We then considered the availability of information about these products, eliminating the ones for which limited information was available. Finally, with the information compiled from the interviews, survey and literature review, the simplified value chains were delineated considering the main actors involved in the process, as well as the feedstock type and source, the type of processing plant, and the targeted product.

## Results

### General interview and survey results

We received 11 responses through the online survey from a pool of 39 stakeholders (28% response rate). We had an additional 22 respondents who participated through the link shared on social media, but only 11 of them contributed with relevant data. Based on information about the participants’ geographical location and the type of industry where they were active, we believe these results are adequate, as our objective was not to have all active organizations participating on the survey, but rather to fill in as much as possible the missing gaps left by the first group of participants.

The distribution of respondents within the EU was quite balanced, with a slightly higher response rate from participants from Germany (15%), Ireland and Finland (13% each). Two of the respondents were representatives from start-ups, seven from small or medium-sized enterprises, eight from multinationals, and five from research institutes.

**Figure 2.**
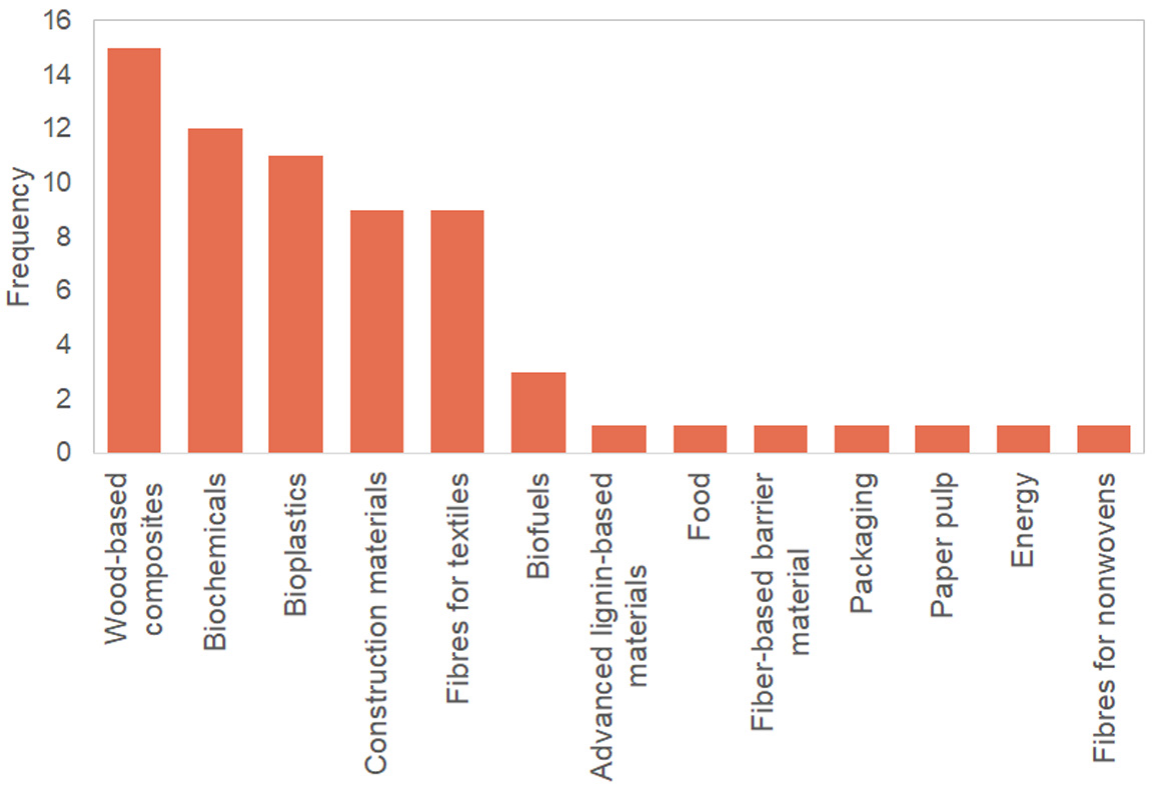
Number of times products were mentioned by interview participants and survey respondents.

During the interviews, the 10 participants mentioned a total of 22 products, covering all five pre-established product categories (i.e., construction materials, chemicals, bioplastics, wood-based composites, and textile fibres). Survey respondents mentioned a total of 45 products, and these were rather balanced across the five product categories. Products mentioned during the interviews and survey that did not belong to any of the five pre-specified categories included biofuels, lignin-based materials, food, fibre-based barrier material, packaging, pulp for paper, energy and fibres for nonwovens. Figure 2 shows the range of products mentioned in the interviews and survey.

From the pool of products mentioned by the survey respondents, 36% were considered final products (e.g., packaging and biofuels). Among the types of feedstocks mentioned by the respondents, regardless of the type of product, wood pulp (22%) and wood chips (20%) were the two most common ones, followed by sawdust (13%) and others (45%). Other types of feedstocks included tree resins and gums, lignin, and recycled wood-based materials, among others.

**Figure 3.**
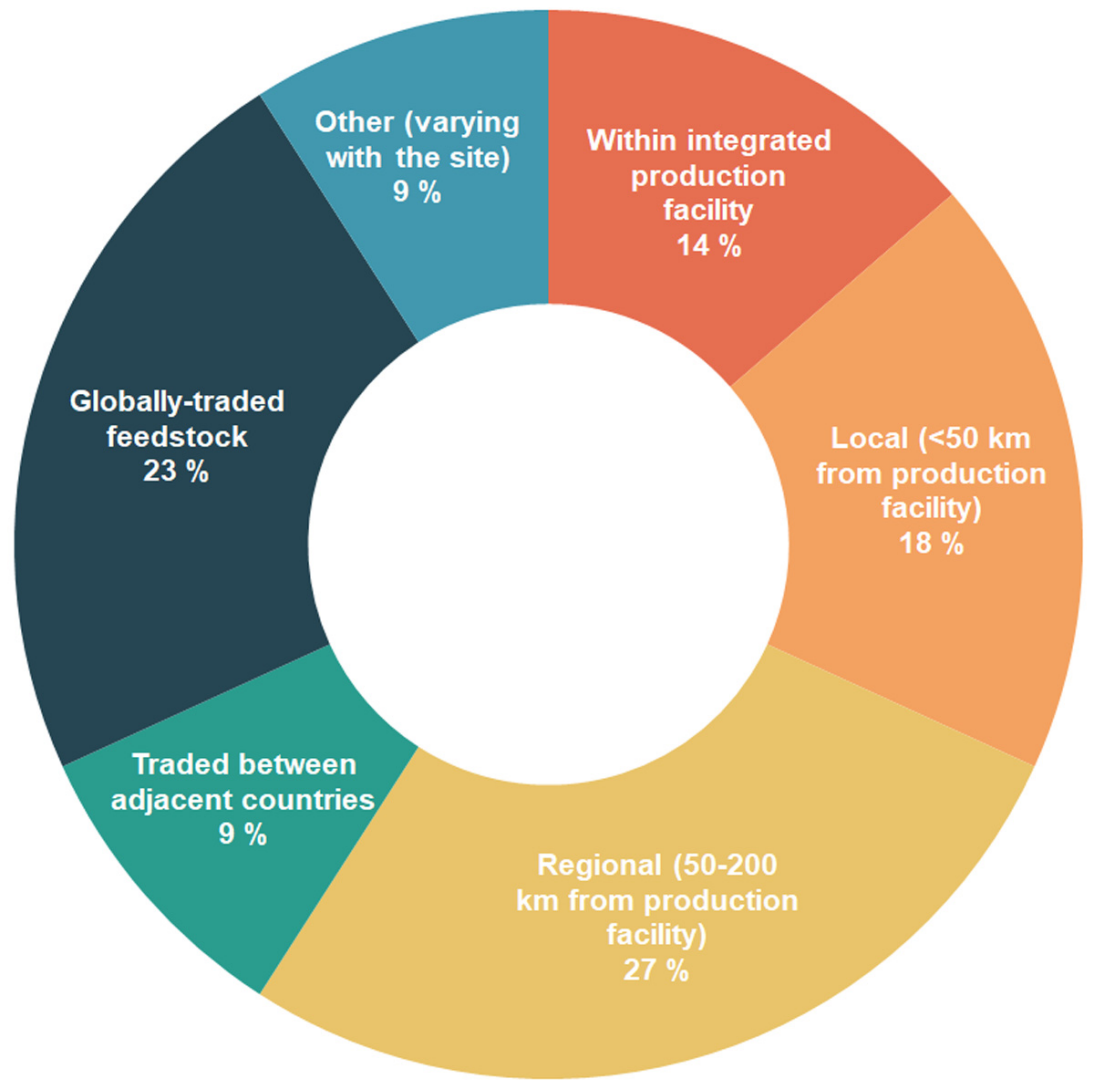
Distance of feedstock source to the processing plant, as mentioned by interview participants and survey respondents.

According to the survey results, respondents indicated they obtained feedstock mostly regionally (50–200 km from production facility) (six respondents), followed by globally traded feedstock (five respondents) ( Figure 3). Some respondents mentioned their feedstock came from local sources (<50 km from production facility, four respondents). For some products, especially the ones developed or produced by companies that worked with a large assortment of products, the feedstock was obtained from within an integrated production facility (three respondents). Few respondents sourced their feedstock from adjacent countries or from varied sources, depending on the site (two respondents, each).

**Figure 4.**
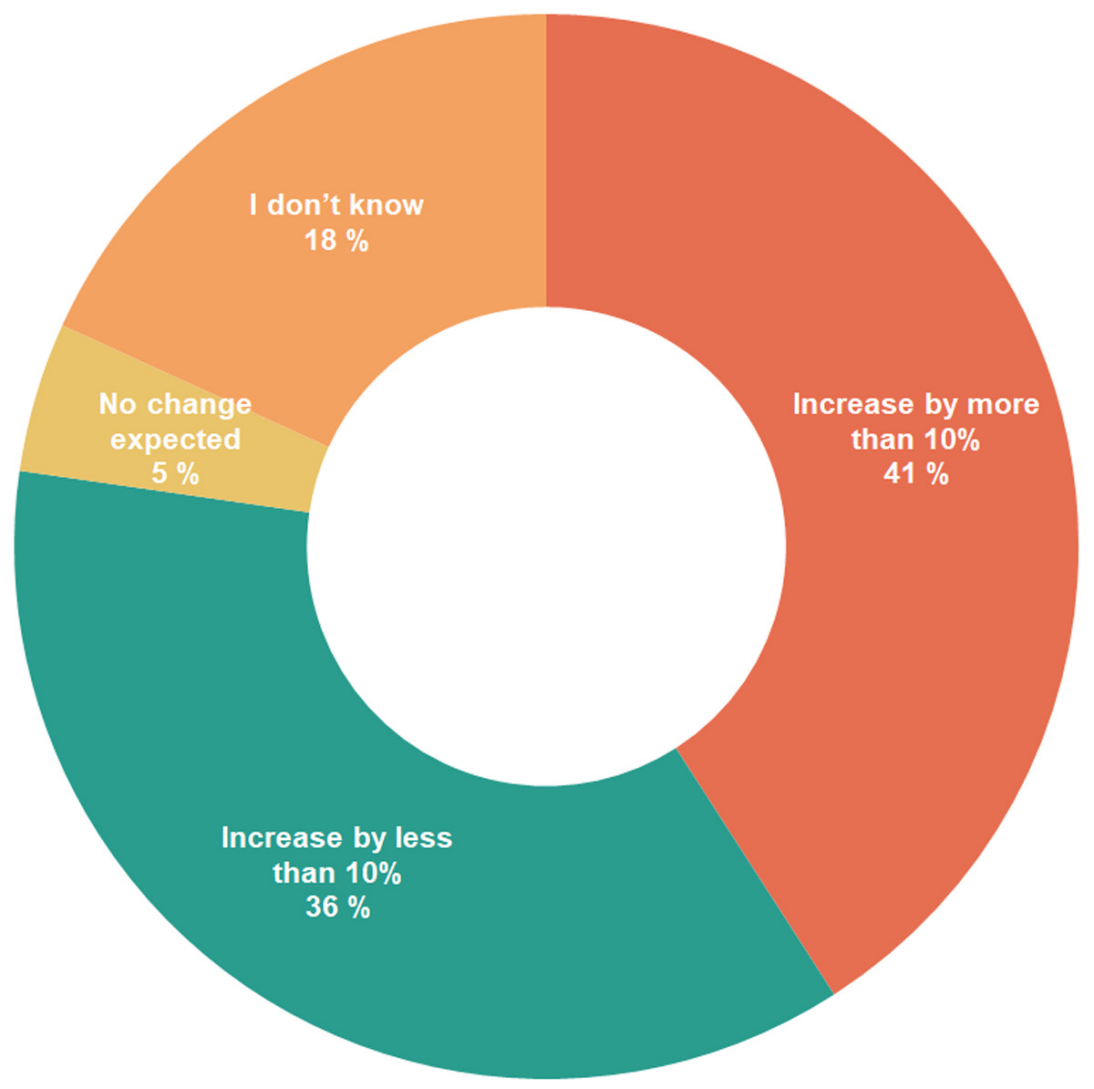
Expected change in demand for biomass.

Regarding the demand for biomass, most survey respondents (77%) expected an increase in demand ( Figure 4). Among these, 41% expected an increase by more than 10%, and 36% expected an increase by less than 10%. The other respondents did not know whether the demand would increase (18%) or expected no change in demand (5%).

When asked if the forest product in question could substitute a fossil-based or GHG-intensive product to some extent in the value chain, 50% of the respondents considered their products to be drop-in. About a third (32%) of the new forest products was considered a partial substitute to fossil-based or GHG-intensive products, and some adjustments would be needed in the value chains. According to the respondents, 4% of the products would need new value chains to be created, and the remaining 14% did not have an answer for the question.

**Figure 5.**
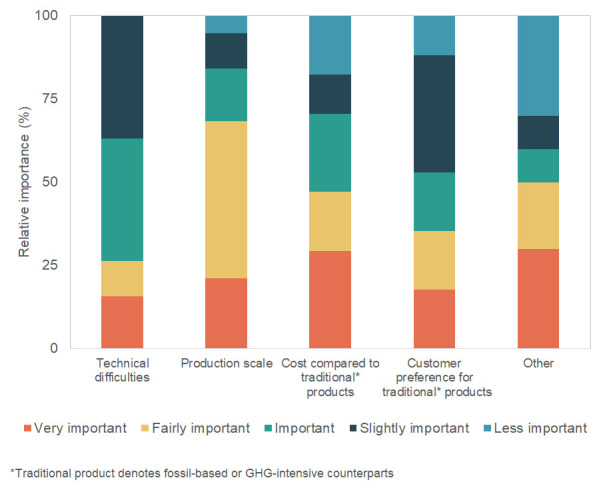
Greatest obstacles for introducing the product to the market or increasing its market share and their importance for the stakeholders.

Among the survey respondents who indicated the greatest obstacles for introducing their products to the market or increasing their market share, 29% mentioned the cost compared to fossil-based or GHG-intensive products as being the most important hindrance ( Figure 5). Other difficulties were also mentioned as the most important aspect, with respondents mentioning as barriers the low feedstock availability, the production scale needed to disrupt the market, and the low availability of venture capital and governmental support. According to the respondents, the second greatest obstacle was related to the shift in production scale from pilot to full scale. Respondents considered technical difficulties an important to slightly important obstacle. Customer preference for traditional (i.e., fossil-based, or GHG-intensive) products was considered only a slightly important obstacle. Other difficulties mentioned by respondents were the high price of raw material, and the supply chain and market development. Among the survey participants, 38% indicated these difficulties could be largely alleviated by EU policies, 33% said that they could be slightly alleviated, and 29% did not know or did not answer the question.

Among the products mentioned by the respondents during the interviews and survey, some were in early stages of development and others had still limited information available on the technology development and production process. According to the ensemble of our data, the most promising products that have been on the market for many years, that had the potential to increase in market share, and that were mentioned by the participants were cross-laminated timber (CLT), wood-based composites and lyocell fibre for textiles. The new products mentioned by respondents that had the most potential to reach the market in the next decade or increase in market size were namely: wood foam, lignin-based adhesives, glycols, bioplastics from ethylene and tall oil, and wood-based textile fibres that use ionic liquid in the production process.

### Innovative forest products

Based on the information obtained during the interviews, survey and literature review, we present, in the following sections, an overview of the selected products organized in three value chains. The first one focuses on the use of sawlogs as input, the second one on the use of pulp logs, and the third one on the use of industrial side streams from sawmills and pulp mills.

**Figure 6.**
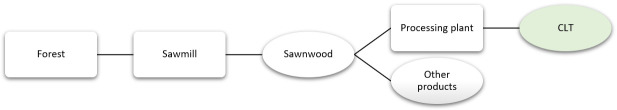
Simplified value chain focused on the use of sawlogs as input, with the reviewed product in green.

***Innovative products using sawlogs as input.*** Among the forest products selected for a detailed analysis, only CLT is produced exclusively from sawlogs. Figure 6 presents a simplified value chain that has CLT as one of the products.

At first, the development and improvement of the technology to produce CLT was rather slow ( Karacabeyli & Gagnon, 2019); however, in the early 2000s, its value chain was developed and the production increased with the interest for new sustainable building materials, product approvals and stronger marketing strategies ( Karacabeyli & Gagnon, 2019). In the past few years, there has been an increase in interest for CLT due to the development of the wood construction sector. It is a solid wood panel suitable for several structural applications, as well as ceilings, floors, and walls ( Anttonen, 2015). Construction with CLT offers many advantages, including fast completion time, low overall weight, resistance and flexibility for construction in earthquake-prone areas ( Anttonen, 2015), the possibility to produce prefabricated elements ( Hurmekoski *et al.,* 2018), and good thermal and fire performance. Because this product allows for a lighter construction, foundations and footers can be built more efficiently and cost-effectively ( UNECE/FAO, 2015).

In the EU, CLT is usually produced using coniferous species, such as spruce, pine, larch, and fir ( Swedish Wood, 2019). The tree species influences the production process (e.g., lamination, bonding), appearance and properties of the final product (e.g., shrinkage and swelling, mechanical resistance). Because CLT is used as a structural element, it is crucial to know the quality of the individual wood components, but also have knowledge on their combined behaviour and the effect of parameters of the manufacturing process on the performance of the final product ( Karacabeyli & Gagnon, 2019).

Regarding the substitution of non-renewable materials by CLT, it can displace concrete, masonry and steel. When compared to equivalent constructions built with steel or concrete, wood-based constructions emit 20–50% fewer GHG, over a 100-year period ( Upton *et al.,* 2008). According to Churkina *et al.* (2020), the construction of timber buildings in urban environments could store from 36.7 to 2495.6 Mt CO _2_·y ^-1^, depending on the scenario and floor area per capita. The biogenic carbon content for CLT at the mill gate is around 762 kg CO _2_e·m ^-^³ (or 207.8 kg C·m ^-^³) ( Stora Enso, 2020). Substituting concrete floor slabs for CLT panels in buildings could contribute to reducing global GHG emissions by on average 50 Mt CO _2_e (excluding carbon sequestration and storage effects), if considering the full uptake of hybrid buildings by 2050 ( D’Amico *et al.,* 2021).

One of the issues that may arise with the use of CLT is related to the environmental performance of the adhesive used in this type of engineered wood product. The adhesive used in the production of CLT is usually formaldehyde-free polyurethane, but phenol-resorcinol formaldehyde and emulsion polymer isocyanate are other commonly used adhesives ( Verkerk *et al.,* 2022). In the past few years there has been an increased interest in developing bio-based options for adhesives to improve the environmental performance of products (i.e., reducing the carbon footprint, reducing toxicity, reducing energy input during production, and improving biodegradability) ( Heinrich, 2019; Siddiqui, 2013).

### Innovative products using pulp logs as input

**Figure 7.**
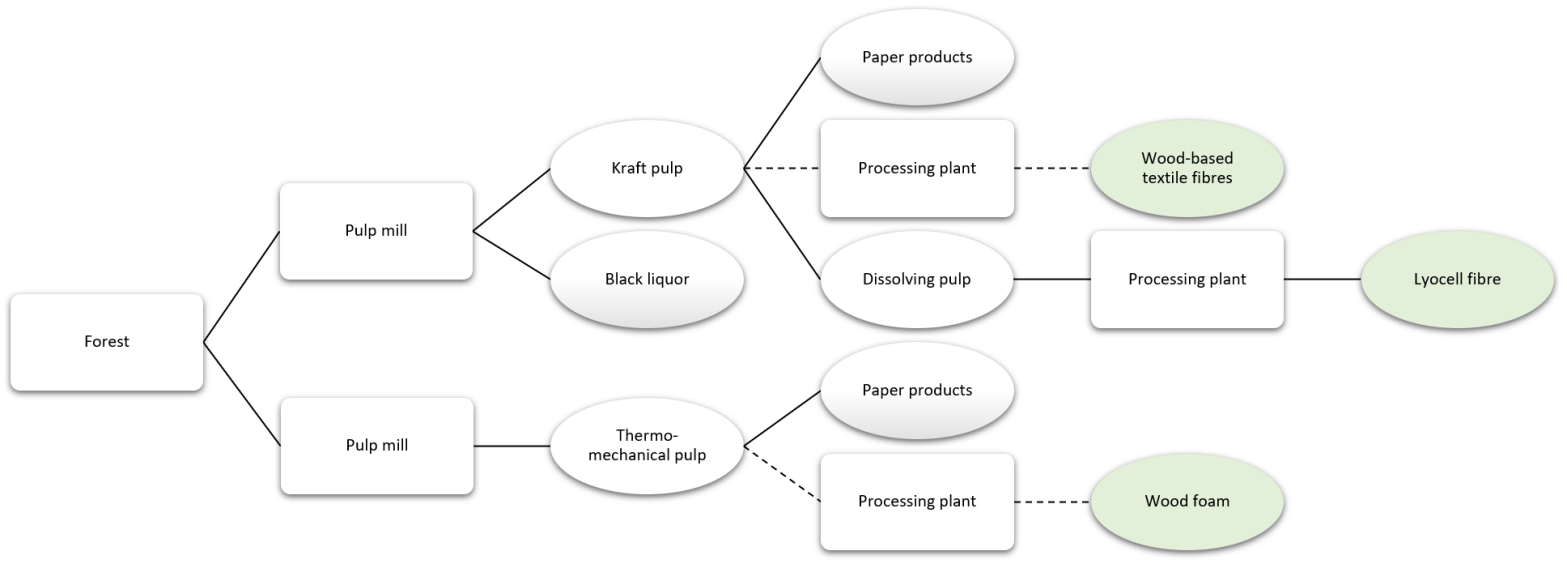
Simplified value chain based on the use of pulp logs and small logs as input for products, with the reviewed products in green. Solid lines denote existing routes and dashed lines represent potential routes.

Many innovative products mentioned by interview participants and survey respondents rely on pulp logs or small logs as primary input material. Figure 7 presents the value chain focused on products obtained from pulp logs or small-dimension logs.

***Wood foam.*** Wood foam is a rigid foam made from cellulose with low bulk density and high insulative capacity. Having a TRL 5–6, it is not yet being produced commercially in any country, but it could become part of the pulp and paper value chain as it uses thermomechanical pulp as feedstock. Wood foam tiles can be used in walls, in the middle of wood panels for furniture and doors, in panels with metal to improve fire resistance properties ( Fraunhofer Institute, 2020; Ritter, 2019a) or with textile-reinforced concrete for lightweight elements ( Ritter, 2019b). Concrete is considered a GHG-intensive material, but adding wood foam to reduce the amount of concrete used in buildings could help reduce CO _2_ emissions of the overall construction project ( Ritter, 2019b). Wood foam can also be used in packaging solutions, as moulded wood foam, substituting polystyrene foam.

Wood foam can be produced from wood fibre supplied in the form of small logs and woody residues from forest operations. It does not contain binders or resins, being therefore free of toxic compounds. Wood foam tiles could possibly substitute materials commonly used in packaging and building construction, such as expanded polystyrene, polyurethane and polyisocyanurate ( Pavel & Blagoeva, 2018). For thermal and acoustic insulation, for instance, wood foam performance is equivalent or better than polystyrene of the same thickness ( Fraunhofer Institute, 2020; Ritter, n.d.). Although wood foam is seen as a potential substitute for polystyrene, polyurethane and polyisocyanurate, information on substitution effects is not yet available for the bio-based material. In addition, unlike these traditional materials, wood foam is fully biodegradable and could be recycled, characteristics that are especially important considering the plastic pollution and its effects.

***Textile fibres.*** From kraft pulp, it is possible to produce not only paper products, but also fibres for textiles (also known as man-made cellulosic fibres). Perhaps the most well-known wood-based textile fibre is viscose, which has been produced for over a century. However, some viscose production processes are very energy-intensive, generate toxic chemical waste ( Sayyed *et al.,* 2019), and cause water and air pollution, among other issues ( Changing Markets Foundation, 2017). While lyocell has some similarities to viscose, such as using dissolving pulp in their production process ( Zhang *et al.,* 2018), it is considered its own type of fibre. Lyocell is produced using a non-toxic solvent (N-methylmorpholine N-oxide) ( Krysztof *et al.,* 2018), of which 99% can be recovered and recycled ( Borbély, 2008). Some new technologies for the production of textile fibres (e.g., Spinnova, Kuura, Ioncell) combine mechanical treatment with non-harmful chemicals (e.g., ionic liquid), without dissolving the wood pulp in any stage of the process. The TRL of new wood-based textile fibres varies from 5 to 9, depending on the production process. These new textile fibres will be part of the existing wood-based fibres value chain, placed together with viscose and lyocell.

Wood-based textile fibres can be produced with many types of biomass. Currently, the most common tree species used for this purpose are birch and eucalyptus, although many other species can also be used (e.g., beech, spruce and pine). According to our survey respondents, approximately 2.5 tonnes of oven-dry wood are required to produce one tonne of cellulosic fibres, depending on the production process. One of the companies that participated in our survey uses about 1.8 million tonnes per year of biomass to produce their textile fibre. Survey respondents expected the volume demands to increase around 10% in the next 10 years.

In general, modern wood-based fibres have a lower environmental impact compared to cotton, viscose, and synthetic fibres such as polyester and polypropylene. The main factors contributing to this are the use of renewable energy during the production process, the use of less chemicals and water, and the lower GHG emissions ( Shen *et al.,* 2010). During the early stages of product development, survey respondents mentioned as important aspects the use of low impact raw materials, the use of optimised production processes to minimise negative environmental impacts, and the design considering recyclability, biodegradability and waste minimization at the end of life.

Depending on the type of textile fibre, they can be fully or partly recyclable. When partly recyclable, the issues are mostly related to the end-use (which influences the type of fibre blends) and the collection systems in place. Wood-based fibres are technically fully recyclable; however, the actual recycled content is likely closer to the textile value chain (i.e., less than 1% recycled for garments, and about 12% for cascade recycling). Filtering is an additional step required during textiles recycling, if the dissolved textiles were previously blended with polyester or other synthetic materials. Regarding the biodegradation of the material, because the new wood-based fibres have a share of bio-based materials of 100%, they are fully biodegradable in water and soil, compostable in commercial or industrial composting facilities, and in some cases at home.

### Innovative products using industrial side streams as input

**Figure 8.**
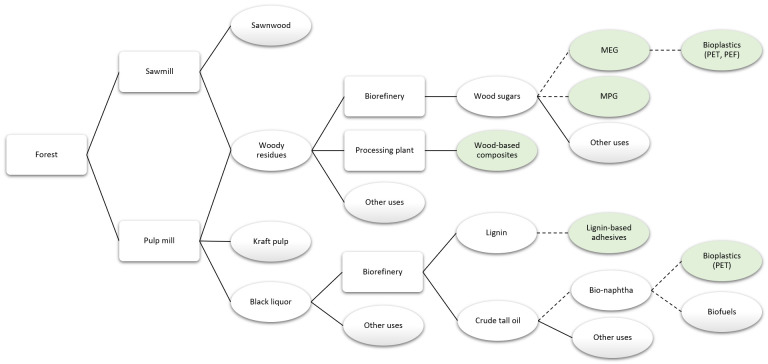
Simplified value chain focused on products that might use industrial sides streams as input, with the reviewed products in green. Solid lines denote existing routes and dashed lines represent potential routes. MEG is monoethylene glycol, MPG is monopropylene glycol, PET is polyethylene terephthalate, and PEF is polyethylene furanoate.

Many products can be manufactured using residues from sawmills and pulp mills, taking advantage of the availability of low-value raw material. Figure 8 presents a simplified value chain focused on products that could use industrial side streams as input.

***Glycols.*** Glycols are a group of chemical compounds widely used in several industries, with ethylene glycol being the simplest type. Ethylene glycols are commonly used in certain types of plastics, in automotive anti-freeze liquids, in adhesives and paints, among other applications ( Forkner *et al.,* 2004). One type of glycol used in the production of polyesters for textiles and packaging is monoethylene glycol (MEG). Currently, 99% of MEG is produced from fossil sources. Regardless of the feedstock source, MEG is an important chemical building block for polyethylene terephthalate (PET) or polyethylene furanoate (PEF) polymers, which are commonly used for bottles and packaging, textile fibres, paint solvents, among others.

Another type of glycol is monopropylene glycol (MPG), which can be used in anti-freeze agents, as a chemical intermediate in the production of unsaturated polyester resins and a solvent used in the manufacturing of detergents. Apart from industrial use, MPG of higher purity has a wide range of uses, including as an additive in cosmetics and personal hygiene and skin care products. Feed-grade MPG can also be used in cattle feed, particularly to avoid ketosis ( Kupczyński *et al.,* 2020).

Most MPG is derived from petroleum, but it can also be produced from plant-based glycerine or from glycerol that can be obtained as a side-product from the production of biodiesel. The environmental impact of bio-based glycerine varies depending on feedstock and production process. As for glycols, the environmental impacts can be attributed to a multitude of factors. The feedstock used for production should be renewable and transport of raw material limited to short distances. An environmental impact assessment comparing petroleum-based and bio-based glycols indicated a burden shifting towards the latter for indicators related to acidification potential, marine and terrestrial eutrophication potential and use of land and biotic resources ( Nachtergaele *et al.,* 2019). This study was based on glycols from agricultural crops and tallow, but hints to issues that may arise with the production of bio-based glycols. Because glycols produced by wood-based industries are still quite new, there is lack of information when it comes to their environmental impacts.

Bio-based glycols can be produced from sugars extracted from agricultural crops, but also from woody biomass, in particular from broadleaved trees such as beech. Thus, glycols made from woody biomass are considered drop-in in the value chain for downstream applications. If demand for this type of feedstock increases, there is a future possibility to do breeding and clone selection aiming for trees with appropriate saccharification properties. Woody biomass would normally be locally sourced, and availability is not predicted to be a problem as there is a variety of possible sources.

Based on the interviews, the substitution potential for replacing petroleum-based raw material for wood feedstock in glycol production could possibly be substantial. At the current capacity, respondents did not expect a decrease in demand for fossil-based glycol sources as the market increases at a rate that surpasses the expected production of wood-based glycol. To the best of the authors knowledge, the most advanced facility aiming to produce glycols from woody biomass is an industrial-scale plant currently under construction in Germany. That would put the TRL level for wood-based glycols at 6–7.

***Bioplastics from wood sugars.*** Ethylene is one of the most important platform chemicals ( Mozaffarian, 2015), which can be used in a myriad of products ( Spekreijse *et al.,* 2019). It is mostly produced from petroleum but can also be obtained from bio-based sources, typically from maize, sugar beet, and sugarcane. Recently, woody biomass has been introduced as a technically feasible feedstock for producing bioplastics. Some companies have started converting wood sugars into MEG for bioplastic films, which can be used as an alternative to fossil-based plastic coating on liquid cartons.

Ethylene is commonly produced from (fossil-based) naphtha, gas oil and condensates, but the bio-based counterpart can be produced through dehydration of bioethanol ( Mozaffarian, 2015). Bioethanol, in turn, can be produced from any type of woody biomass, as its main component is cellulose, which is primarily composed of the sugar ‘glucose’. Other types of fermentable sugars (e.g., xylose and mannose) can also be found in wood and could be used as sources for ethanol. In addition, ethanol can also be produced using certain types of bark as feedstock ( Reina *et al.,* 2016). Therefore, the feedstock to produce bioplastics using the ethylene route could be any type of wood residues. Based on the responses from the survey, the wood used to produce wood sugars comes mostly from regional sources, and at times from local or globally traded sources.

Estimating the quantity of woody biomass to produce one unit of ethylene-based bioplastic is not straightforward as there are many factors that influence on the product yield. Some examples of factors affecting the volume of ethylene-based bioplastics are: the tree species and part of the tree used as raw material, the process used to extract the sugars, and the chosen production process for ethanol and ethylene, among others. To illustrate the variations due to some of these factors, the ethanol yield obtained from woody biomass may vary from 2% on unbleached pine pulp ( Przybysz Buzała *et al.,* 2017) to 53% on unbleached kraft pulp of eucalyptus ( Branco *et al.,* 2020). It is important to remember that the conversion of wood to ethanol is only one part of the complex manufacturing process of ethylene-based bioplastics.

Several kinds of fossil-based plastics can be replaced by bioplastics, including PET and polyurethane ( European Bioplastics, 2020). Bioplastics can be a solution to some of the current problems stemming from traditional plastics, such as the dependency on fossil sources of feedstock and the increased GHG emissions ( Ferreira-Filipe *et al.,* 2021). The use of renewable feedstock is one of the benefits of bioplastics production connected to the forest sector, especially when using waste and industrial side streams. Another advantage is the reduced emission of GHG associated with the production and use of the raw materials ( Mozaffarian, 2015). However, the production of bioplastics from side streams of the wood industry is technically challenging and frequently economically unviable ( Brodin *et al.,* 2017). From the perspective of the forest sector, there is a need for a new value chain to produce these chemicals and bioplastics.

One possible issue with bioplastics is related to the end-of-life options for the material. While using biomass as feedstock may help solve issues related to the dependance of non- renewable resources, it may not necessarily contribute to reducing plastic pollution, as bioplastics are not necessarily biodegradable, compostable or recyclable ( Maier, 2018; Tenhunen & Pöhler, 2020). For this reason, it is important to take sustainability aspects into consideration during product conception and manufacture and have adequate disposal of the material at the end of its life to avoid pollution, especially caused by microplastics ( Neves *et al.,* 2020). It is also important to mention that currently many bioplastics are only partially bio-based. Bio-PET, for instance, is produced from ethylene glycol from biomass. However, it also requires terephthalic acid in the production process, which is only available commercially from fossil sources, resulting in a bio-PET that is approximately 30% bio-based ( Spekreijse *et al.,* 2019).

***Lignin-based adhesives.*** Lignin is a by-product of the pulp and paper industry, most of which is currently combusted for internal energy production ( Hu *et al.,* 2011). Yet, it is a suitable raw material for carbon fibres ( Souto *et al.,* 2018), pharmaceutical materials ( Gil-Chávez *et al.,* 2019), 3D printing composites ( Yu & Kim, 2020), adhesives ( Alinejad *et al.,* 2019; Li *et al.,* 2018), chemical building blocks and platform chemicals ( Neis-Beeckmann, 2017; Wong *et al.,* 2020), among others. One of these chemical building blocks is catechol, which may be used as a substitute of resorcinol in formaldehyde resins ( Neis-Beeckmann, 2017). Because lignin is the most abundant natural phenolic polymer, it could also be used as a substitute for phenol in phenolic adhesives ( Luo & Shuai, 2020; Kalami *et al.,* 2017). The TRL varies according to the production process and the type of end-product. One of the lignin-based phenolic adhesives that can be found in literature had an estimated TRL of 8 ( University of Bologna and Fraunhofer ISI, 2018). The development of lignin-based adhesives is mostly connected to the pulp and paper industry. Some adjustments will be needed to the value chain to include biorefineries that can fractionate the black liquor into value-added chemicals to produce lignin-based adhesives.

Estimates of lignin availability vary between 50–100 million tonnes per year ( Bajwa *et al.,* 2019; Neis-Beeckmann, 2017). One respondent indicated they produce 50 thousand tonnes of lignin each year in a single mill. However, from the global lignin production, less than 5% are used for value-added purposes, such as phenolic resins, foams, and surfactants ( Bajwa *et al.,* 2019; Hu *et al.,* 2011). A lot of effort has been put into developing lignin-based adhesives to substitute fossil-based phenolic compounds and take advantage of the large availability of lignin ( Hu *et al.,* 2011) and its lower price compared to fossil-based phenol ( University of Bologna and Fraunhofer ISI, 2018). There are many processes for recovering lignin from black liquor with quality that would be suitable for phenolic resins and polyurethane foams ( University of Bologna and Fraunhofer ISI, 2018). Among the several sources of lignin, the best fossil-based phenol substitute is the one from pine obtained through the kraft pulping process ( Tejado *et al.,* 2007).

The adhesive used in wood panels and engineered wood products has a lot of influence on their environmental performance, particularly when it comes to the source of feedstock, emissions of volatile organic compounds during the use stage, and product disposal at the end of the life cycle ( Messmer, 2015). Anther advantage of substituting fossil-based phenolic resins by their lignin-based counterparts is the lower use of energy during production ( Siddiqui, 2013). Even though it is not yet cost-effective to produce engineered wood products with lignin-based adhesives, using industrial lignin as a component in the adhesives can achieve good results by reducing the amount of synthetic phenol needed ( Hemmilä *et al.,* 2017; Nakos *et al.,* 2016).

***Bioplastics from tall oil.*** Besides being the precursor to lignin and its derivatives, the black liquor resulting from the pulping process can also yield tall oil. Crude tall oil can be fractioned into several chemical compounds, including bio-naphtha, which can be used in the production of biodiesel and bioplastics ( De Bruycker *et al.,* 2014). In addition, crude tall oil can be used to produce, among other chemicals, ethylene and the bioplastics produced from this chemical compound ( De Bruycker *et al.,* 2014). The production of bioplastics from tall oil would be connected to a certain extent to the pulp and paper industry. However, adjustments are needed to the value chain to include biorefineries that can fractionate the black liquor from the pulping process and produce bio-naphtha from crude tall oil.

According to the survey respondents, to produce bioplastics their companies mostly use feedstock from regional sources, and sometimes from local or globally traded sources. One large manufacturing company sources woody residues from sustainably managed forests near the processing plant.

One type of bioplastics that can be produced from bio-naphtha is PET, which is technically equivalent to the same material produced from fossil sources ( European Bioplastics, 2020). Therefore, bio-based PET can be used for food packaging without changes in legislation. It can also directly substitute polyurethane ( European Bioplastics, 2020). One of the advantages of bioplastics from the forest-based sector is using renewable raw materials, industrial side streams and waste. In addition, GHG emissions during product manufacture are lower than fossil-based plastics ( Mozaffarian, 2015). Some bioplastics from tall oil, such as the barrier films for liquid packaging, can be recycled with paperboard, which helps improve the circularity of these products. A constraint for the development of this segment would be the availability of the feedstock, as tall oil is already used for many bio-based products ( De Bruycker *et al.,* 2014). Disadvantages related to this material are related to the end-of-life options for the product, as previously mentioned for bioplastics from wood sugars.

***Wood-based composites.*** Products that combine small wood elements, such as particles and chips, with a binding agent or thermoset polymer are called wood-based composites or wood-thermoplastic composites. These products have been used for many decades as construction material (in decking, siding, roofing among others), but have recently gained more applications due to new technologies and process improvement. The value chain for the new wood-based composites has already been developed, at times being adapted from traditional value chains. Due to concerns about the sustainability of resources and aiming to limit the use of plastics in products, companies have started investing in wood-based composites with a higher proportion of bio-based raw materials, that are mechanically recyclable and biodegradable. Fully biodegradable binders or bio-based binders, such as polypropylene or polylactide, are used in some of these new wood-based composites ( Mäntyranta, 2020).

Several sizes of wood elements can be used to produce composites, such as solid wood pieces, wood chips, sawdust, and wood fibres, as mentioned by survey respondents. According to one participant, 400–700 kilograms of woody feedstock are required to produce one cubic metre of wood-based composite. Most companies developing or producing wood-based composites that participated in our study mentioned sourcing their feedstock from regional or local sources. One of the respondents mentioned that less than 10% of the biomass used each year for the composite material is imported. The respondents expected the volume demands of wood biomass used to produce the composites to increase (either slightly or considerably) in the coming 10 years.

Plastic products can be substituted by durable wood-based composite products (e.g., countertops, furniture, containers) and disposable wood-based composite products (e.g., beverage straws, cotton swab sticks). Similarly to bioplastics, the ban on plastic, polystyrene packaging, and other single-use products has boosted the development of these new wood-based composites ( UNEP, 2018). Although they are considered drop-in, survey respondents mentioned that this material could substitute from 10% to over 90% of fossil-based or GHG-intensive materials, depending on the type of product and its end-use.

Bio-based materials can play two roles in wood-based composites. They can be used as reinforcement and fillers, reducing the share of fossil carbon and increasing the proportion of renewable carbon in the products, or they can be in the form of binders, substituting fossil-based plastics and resins and increasing the share of bio-based carbon. As an example, a wood-based composite intended for durable, waterproof products, has a lower carbon footprint than a technically equivalent ceramic product. According to a lifecycle assessment (LCA), the carbon footprint of the whole product lifecycle is 55 kilograms lower per unit than the ceramic counterpart ( Nurmio, 2018). Survey respondents stated that the share of bio-based materials in their products was above 76%.

According to survey respondents, certain aspects were important during the main product development, such as the use of low impact raw materials and optimised production processes to minimise negative environmental impacts; the design for ease of maintenance, reparability, upgradability and adaptability; and the design considering recyclability, biodegradability and waste minimization at the end of life. In some cases, wood-based composites can significantly reduce energy and natural resource consumption, as well as waste generation and GHG emissions. At the end of the lifecycle, depending on the type of wood-based composite, they can be mechanically or chemically recycled. Others are fully biodegradable in water and in soil, not releasing any microplastics in the environment, and compostable following the standard EN 13432, which requires the material to fully biodegrade in less than 12 weeks. One of the survey respondents mentioned that one type of composite was not compostable, but still had the option of being at least partly recycled.

## Discussion

In this study we identified and reviewed innovative forest products in the EU, covering a wide range of products. We found a rich set of intermediate products with many potential end-uses, as well as end-products in a variety of categories. The current diversity of products is extremely wide: from molecules to bioplastics and composites to large items such as building materials. The growth of these companies and their increasing share of the market in their specific segment could form a significant force in EU bioeconomy and should be treated as such.

As demands for materials such as chemicals and plastics are rising, we could not expect a decrease in demand for fossil-based products in the short term, but the substitution potential is correspondingly large. This also means that the need for woody biomass is expected to increase, a thought that was stressed by most respondents in the survey. Most products covered in this study do not have specific demands on biomass quality, and many can also be produced from what would normally be assumed as waste from the forest industry. In some cases, specific feedstock properties could be beneficial but are not essential. Most selected products can be considered drop-in in relevant value chains. Our selection criteria have most likely favoured drop-in products as we were aiming for market or close-to-market products. Products that would require new or modified value chains have a higher threshold to market introduction at a relevant scale.

As a part of the bioeconomy, the demand for woody biomass varies depending on the product category. For instance, with the boost in construction of multistorey wood buildings around the world, the demand for wood suitable to produce structural elements will most likely increase. For biochemicals and bioplastics, the complex chemical composition of wood makes it a less attractive source than short rotation crops, although there is a breeding opportunity to produce wood better suited for sugar-based production platforms ( Escamez *et al.,* 2017; Gandla *et al.,* 2018). However, with future demands for food production, the use of agricultural land to grow biomass for industrial use is less likely to be considered sustainable. Thus, the use of woody biomass for a diverse range of products could be an alternative that does not compete with food and feed production. Furthermore, forestry is generally a low environmental impact production system, as in Europe, no or limited amounts of fertilizers and pesticides are used, it can prevent soil erosion in contrast to agriculture, and the processing of the material has several potentially valuable side streams. However, breaking down the wood to usable components is a more energy-consuming process than to using agricultural crops. It is reasonable to conclude that the market demand for biomass will increase and that wood will have an environmental edge over annual crops due to non-competing use of land with food and feed, even more so when considering the use of industrial side streams.

Woody biomass can be used for a very broad variety of products other than traditional wood products such as lumber and paper. Firstly, the pulping process generates side streams that can be used to lessen the waste from pulp mills. Residues are often used for internal energy consumption within the sector but are now increasingly processed for other products. For instance, lignin can be extracted from black liquor and is the raw material for many novel bio-based products such as adhesives, material for batteries and resins. Additionally, many products can use woody biomass that could be considered waste (i.e., sawdust, branches or wood types that are normally not used industrially). For instance, several survey respondents named sawdust as raw material for their products. Potentially, an increasing demand for sawdust could make access to the material a limiting factor, especially since sawdust is also a source for bioenergy (frequently in the form of wood pellets).

Based on the product segments that bio-based materials can enter to substitute fossil-based products, the potential market for forest products is quite extensive. Market segments such as chemicals are not only responsible for GHG emissions, but also occur at a very large scale. Even with new forest products coming into the market, stakeholders predict that a very small proportion of traditionally manufactured chemicals will be substituted in the coming 10–20 years. This means that customer demand for renewable alternatives combined with EU policies favouring forest products, the drive for increasing production will be quite strong, and consequently the increase in biomass needs will be high.

In a bioeconomy context, one must take into consideration the full lifecycle of products. This means that not only the source of raw material is important but also the ratio of bio-based materials in the product, the energy consumption for manufacturing, the residues produced, and the product’s end-of-life. Almost all forest products covered in our survey were recyclable to some extent. Many were also biodegradable, which indicates an environmental advantage, even if the results from full LCAs are not available. It is also clear that the environmental impact is an important factor when working with these products. Most of the survey respondents mentioned taking into consideration sustainability measures during product development and for many products at least a cradle-to-gate LCA was conducted.

In general, companies do experience difficulties in introducing their products in the market or increasing the market share. They believe these obstacles can be partly alleviated by EU policies. The level and type of support required differ. However, survey respondents believe less bureaucracy, support for pilot-scale to full-scale production and subsidies for bio-based alternatives can help alleviate these difficulties.

## Conclusions

There is a profusion of innovative forest products at different stages of development and manufacture, although most products can likely not be technically and economically produced nor have the potential to increase the market share. According to our findings, the novel products that are most likely to increase in market size are CLT and wood-based textile fibres. Among the new products, wood foam, glycols, bioplastics (both from tall oil and wood sugars), lignin-based adhesives and wood-based composites are the ones that have potential to enter the market in the next 20 years.

With these forest products entering the market or gaining more popularity, the demand for woody biomass is expected to increase in the future. While feedstock quality is not a crucial factor for most of the reviewed products, some could benefit from forest management strategies such as tree breeding. Other types of feedstocks, which are normally used for energy purposes in the industry (e.g., sawdust, black liquor), could become a limiting factor in the manufacture of certain innovative products, as it would result in an increased competition by different industries for the same material. Despite this, the use of woody biomass for products could be justified by having the benefit of storing carbon for longer periods in products and materials when compared to carbon being released in the atmosphere when used for energy purposes.

Besides contributing to decreasing GHG emissions and storing carbon, the innovative forest products can also tackle other environmental issues typically associated with their non-wood counterparts, such as reducing pollution and waste generation, and decreasing the competition for land with food and feed production, as is the case for the production of cotton and other non-food agricultural crops. However, as most of these products are still in development or have recently entered the market, it is important to support these claims with third-party assessments, such as LCAs. It would be important to take into consideration the products’ full lifecycle, including the feedstock source, the ratio of bio-based component in the product, the energy consumption for manufacturing, the residues and waste produced, and the product’s end-of-life. Having the environmental benefits clearly demonstrated could potentially increase the uptake of bio-based products, considering the growing awareness from consumers on environmental issues.

Finally, the ease of market introduction of innovative products relies heavily on the products’ ability to take advantage of existing value chains. In general, many products reviewed in this study are considered drop-in, which is an advantage regarding market introduction. This is because products that require adjustments to production lines or methods are less likely to get into the market without strong external drivers that push for bio-based alternatives. Thus, the economic viability and the market expansion of forest products could be encouraged to a certain extent by EU policies. Other measures that could possibly contribute to alleviating the difficulties encountered during the development and manufacture of forest products are reducing bureaucracy, increasing the support for pilot-scale to full-scale production, and increasing subsidies for bio-based alternatives.

## Data availability

Zenodo: Innovative forest products in the circular bioeconomy: online survey questionnaire and dataset, https://doi.org/10.5281/zenodo.5902552 ( Hassegawa *et al.,* 2022a)

This project contains the following underlying data:

- Data_survey_Innovative_forest_products.csv (dataset from online survey)
- Questionnaire_survey_Innovative_forest_products.pdf (questionnaire for online survey)

Data are available under the terms of the Creative Commons Attribution 4.0 International license (CC-BY 4.0).

Zenodo: Innovative forest products in the circular bioeconomy: questionnaire for semi-structured interview. https://doi.org/10.5281/zenodo.5898055 ( Hassegawa *et al.,* 2022b)

This project contains the following underlying data:

- Questionnaire_interview_Innovative_forest_products.pdf (questionnaire for semi-structured interview)

Data are available under the terms of the Creative Commons Attribution 4.0 International license (CC-BY 4.0).
