# Supplementary material for: Innovative forest products in the circular bioeconomy
Source: Open Res Eur. 2022 Jun 16;2:19. Originally published 2022 Feb 9. [Version 2] doi: 10.12688/openreseurope.14413.2 (PMC10445822; doi:10.12688/openreseurope.14413.2)
Supplement: Peer review history

# Peer review: Reviewer response for version 2

**DOI:** 10.21956/openreseurope.16093.r29540

**Published:** 2022-06-21

**Authors:** Michela Nocetti

**Affiliations:** 1 CNR-IBE, Institute of Bioeconomy, National Research Council, Sesto Fiorentino, Italy

I have no further comments.

Is the study design appropriate and does the work have academic merit?

Yes

Is the work clearly and accurately presented and does it cite the current literature?

Yes

If applicable, is the statistical analysis and its interpretation appropriate?

Not applicable

Are all the source data underlying the results available to ensure full reproducibility?

Yes

Are the conclusions drawn adequately supported by the results?

Yes

Are sufficient details of methods and analysis provided to allow replication by others?

Yes

Reviewer Expertise:

Characterization of wood and wood-based products, mostly for structural uses.

I confirm that I have read this submission and believe that I have an appropriate level of expertise to confirm that it is of an acceptable scientific standard.

# Peer review: Reviewer response for version 2

**DOI:** 10.21956/openreseurope.16093.r29539

**Published:** 2022-06-17

**Authors:** Pierre Blanchet

**Affiliations:** 1 Université Laval, Québec, Canada, Québec City, Canada

This version of the paper is acceptable. Great work by the authors.

Is the study design appropriate and does the work have academic merit?

Partly

Is the work clearly and accurately presented and does it cite the current literature?

Yes

If applicable, is the statistical analysis and its interpretation appropriate?

Not applicable

Are all the source data underlying the results available to ensure full reproducibility?

Partly

Are the conclusions drawn adequately supported by the results?

Yes

Are sufficient details of methods and analysis provided to allow replication by others?

Partly

Reviewer Expertise:

Wood construction, product development, sustainable buldings, qualitative and quantitative research

I confirm that I have read this submission and believe that I have an appropriate level of expertise to confirm that it is of an acceptable scientific standard.

# Peer review: Reviewer response for version 1

**DOI:** 10.21956/openreseurope.15557.r28690

**Published:** 2022-03-31

**Authors:** Pierre Blanchet

**Affiliations:** 1 Université Laval, Québec, Canada, Québec City, Canada

The paper is interesting, especially on a short-term perspective. This being said, there are a few things to improve.

 First, the authors mention in their methodology the use of triangulation and are referring to Tassinari *et al* 2021. This paper does not refer to the triangulation methodology, I invite them to consider the paper of Mathison, S. (1988). "Why triangulate?," *Educational Researcher *17(2), 13-17 ^1^ and Patton, M. Q. (1999) ^2^. Enhancing the quality and credibility of qualitative analysis. *Health services research*, 34(5 Pt 2), 1189.

 On the triangulation, in the method section, limited details are provided, so we cannot understand what was the state of mind to make their three information pillars into observations. More details should be provided.

 When describing the survey, they mention 39 stakeholders surveyed but to assess the statistical representation of these we need to know 39 out of how many?

 In the survey section, 10 respondents are mentioned where it is 11 in the results section. Which one is the right one?

 In the product categories, some may overlap, i.e. construction material vs wood composites vs fibres for nonwoven. A nonwoven insulation material is a construction material and a composite as most of them are bonded with polyester. This must be clarified.

 Finally, the authors are suggesting great graphs to support their discussion. The conclusion is clear and concise.

Is the study design appropriate and does the work have academic merit?

Partly

Is the work clearly and accurately presented and does it cite the current literature?

Yes

If applicable, is the statistical analysis and its interpretation appropriate?

Not applicable

Are all the source data underlying the results available to ensure full reproducibility?

Partly

Are the conclusions drawn adequately supported by the results?

Yes

Are sufficient details of methods and analysis provided to allow replication by others?

Partly

Reviewer Expertise:

Wood construction, product development, sustainable buldings, qualitative and quantitative research

I confirm that I have read this submission and believe that I have an appropriate level of expertise to confirm that it is of an acceptable scientific standard, however I have significant reservations, as outlined above.

## References

[1] : Why Triangulate?. *Educational Researcher* .1988;17(2) : 10.3102/0013189X017002013 13-17 10.3102/0013189X017002013

[2] : Enhancing the quality and credibility of qualitative analysis. *Health Serv Res* .1999;34(5 Pt 2) :1189-208 10591279PMC1089059

# Response

**Published:** 2022-06-10

**Authors:** Mariana Hassegawa

**Affiliations:** European Forest Institute, Finland

*The authors thank the reviewer for the time dedicate to review this manuscript and for the valuable feedback. We have tried to respond as best as possible to the points raised by the reviewer. Our responses are shown in italics, and changes made to the text of the manuscript are presented in this document underlined.*

 The paper is interesting, especially on a short-term perspective. This being said, there are a few things to improve. First, the authors mention in their methodology the use of triangulation and are referring to Tassinari et al 2021. This paper does not refer to the triangulation methodology, I invite them to consider the paper of Mathison, S. (1988). "Why triangulate?," Educational Researcher 17(2), 13-171 and Patton, M. Q. (1999)2. Enhancing the quality and credibility of qualitative analysis. Health services research, 34(5 Pt 2), 1189. On the triangulation, in the method section, limited details are provided, so we cannot understand what was the state of mind to make their three information pillars into observations. More details should be provided.

***Response from the authors:*** *Tassinari et al. (2021) point out to the importance of triangulation in studies such as ours, and for that reason their publication was referred to in our text. Nevertheless, and following the reviewer’s suggestion, we have consulted the works of Mathison (1988) and Patton (1992) on triangulation, and included more information about the selection of the three information pillars, with a brief explanation on why we chose each of those methods.* *“Information on the development and characteristics of new or emerging products and technologies is not often publicly available. Therefore, we collected qualitative information and quantitative data using a combination of data collection methodologies, including interviews, a survey, and a scientific and grey literature review. Firstly, the semi-structured interviews would allow us to obtain more detailed answers from the participants (de Leeuw, 2008) and go deeper into discussions about products and aspects that were deemed more important to the organizations developing the products. Secondly, the online survey provided participants the flexibility to respond at their own time and pace, allow for anonymity, and reach a larger and more diverse group of participants (Braun et al., 2020). Thirdly and finally, the literature review allowed us to obtain specific information about products that were mentioned during the study. These multiple methods of data collection would help reduce the errors associated with using a single method (Patton, 1999), by allowing us to triangulate the data from these three sources (Tassinari et al., 2021), examine the data for consistencies and discrepancies (Mathison, 1988; Barnum, 2021), and increase the robustness of our results. A thematic analysis was performed to the qualitative data (Bengtsson, 2016). The COREQ guidelines (Tong et al., 2007) were adopted to throughout this study. The procedure adopted in each of these methods is described in the sections that follow.”* 

 When describing the survey, they mention 39 stakeholders surveyed but to assess the statistical representation of these we need to know 39 out of how many?

***Response from the authors:*** *The 39 stakeholders were part of the list of organizations described in the Methods section, in the sub-section “Scope of the analysis”. We sent an email with the link to the online survey to the 39 stakeholders, but we obtained only 11 responses (28% participation rate), which we deemed too low. To increase the number of responses, we shared the link to the online survey on social media. We were able to obtain 11 additional responses with relevant data, but the representativeness cannot be estimated due to the unknown size of the population (i.e., organizations that use social media). Based on the participants’ geographical location and the type of industry where they were active, we considered the number of survey responses was adequate, as we aimed to fill in as much as possible the missing gaps left by the first group of participants. This information has been added in the results.* *“We received 11 responses through the online survey from a pool of 39 stakeholders (28% response rate). We had an additional 22 respondents who participated through the link shared on social media, but only 11 of them contributed with relevant data. Based on information about the participants’ geographical location and the type of industry where they were active, we believe these results are adequate, as our objective was not to have all active organizations participating on the survey, but rather to fill in as much as possible the missing gaps left by the first group of participants*.”

 In the survey section, 10 respondents are mentioned where it is 11 in the results section. Which one is the right one?

***Response from the authors:*** *The typo was on the survey section; the number of respondents was 11. This has now been corrected.* 

 In the product categories, some may overlap, i.e. construction material vs wood composites vs fibres for nonwoven. A nonwoven insulation material is a construction material and a composite as most of them are bonded with polyester. This must be clarified.

***Response from the authors:*** *Thank you for pointing that out. Indeed, there is some unavoidable overlap in the product categories. Following the reviewer’s comment, we tried to clarify in the “Scope of the analysis” section the types of products that would be included or excluded from the categories with notable overlap.* *“Because of the overlap between some product categories, we considered that “construction materials” would encompass elements used for structural (e.g., engineered wood products) and non-structural purposes (e.g., insulation materials) in buildings. We grouped products such as fibreboards and laminated veneer lumber, which are frequently denominated “composite materials” for being composed of wood elements bonded with adhesives (Stark and Cai, 2021), in the category “construction materials” due to their applications. The category “composites” would include all products where small wood elements (i.e., wood chips, particles, fibres, and woodmeal) are bonded with a binding agent to form products that are purposes other than building construction. “Textiles” would encompass staple fibres and filaments, as well as threads, yarns and fabrics made of wood-based fibres, typically used for clothing and household items. Products that contained textiles combined with other materials, such as cement, foam, resins, etc., were not included in this category for being considered composite materials. The category “chemicals” focused on chemical compounds produced from biomass or through a bioprocessing route (Philp et al., 2013). Cellulose, which is technically a chemical compound, was not included in this category as it is a fundamental wood component. However, chemicals produced from cellulose (e.g., platform chemicals, were considered potential products in our study (Geboers et al. 2011; Nitzsche et al. 2021; Takkellapati et al. 2018). Finally, “bioplastics” comprised products and materials made of bio-based polymers from woody biomass and its derivatives that could be shaped by flow (Vert et al., 2012).”* 

 Finally, the authors are suggesting great graphs to support their discussion. The conclusion is clear and concise. **References** 1. Mathison S: Why Triangulate?. *Educational Researcher*. 1988; **17 **(2): 13-17 Publisher Full Text 2. Patton MQ: Enhancing the quality and credibility of qualitative analysis. *Health Serv Res*. 1999; **34** (5 Pt 2): 1189-208 PubMed Abstract

***Other changes to the manuscript** Besides minor changes to the manuscript, the results presenting the obstacles for introducing the products to the market were revised and updated:* *“Among the survey respondents who indicated the greatest obstacles for introducing their products to the market or increasing their market share, 29% mentioned the cost compared to fossil-based or GHG-intensive products as being the most important hindrance (Figure 5). Other difficulties were also mentioned as the most important aspect, with respondents mentioning as barriers the low feedstock availability, the production scale needed to disrupt the market, and the low availability of venture capital and governmental support. According to the respondents, the second greatest obstacle was related to the shift in production scale from pilot to full scale. Respondents considered technical difficulties an important to slightly important obstacle. Customer preference for traditional (i.e., fossil-based, or GHG-intensive) products was considered only a slightly important obstacle. Other difficulties mentioned by respondents were the high price of raw material, and the supply chain and market development. Among the survey participants, 38% indicated these difficulties could be largely alleviated by EU policies, 33% said that they could be slightly alleviated, and 29% did not know or did not answer the question.”*

# Peer review: Reviewer response for version 1

**DOI:** 10.21956/openreseurope.15557.r28635

**Published:** 2022-03-18

**Authors:** Michela Nocetti

**Affiliations:** 1 CNR-IBE, Institute of Bioeconomy, National Research Council, Sesto Fiorentino, Italy

The article titled “Innovative forest products in the circular economy” aims to give an overview of the more or less innovative bio-based products coming from the forests, which could help to tackle climate change. It is well written and organized, sound and logical in its methodology and exposition of results.

 It seems useful to have an overview of the actual level of development for the bio-based products coming from the processing of the forest raw resources. In some parts, it is a bit too general (wood-based components and biochemical), but it is most likely due to the high number of different products to be included in these categories. However, the literature is up-to-date and comprehensive, for further information.

 Therefore, most of my comments in the following aim to improve the readability of the work.

Introduction

 The sentence “Because of these concerns, the European Union (EU) has banned the production and consumption of single-use plastics (European Parliament and Council 2019), which would also have implications on bioplastics that are technically equivalent to fossil-based plastics (Brodin et al., 2017).” means that the same statement of the European Commission is valid both for fossil-based and bioplastic? The citation is not present in the list of references, by the way.

 About the aims: “To which extent are these forest products compatible with existing value chains?”: as one of the demands of the research, it appears not always clearly stated along with the description of the several products.

Methods

 As concerns the interviews, 12 organizations were selected from a pool of 39 stakeholders. But the 10 interviews were few and the authors decided to go with an online survey. Why they did not try with the interview of the all 39 stakeholders?

 About the survey, again 39 stakeholders are cited. Are they the same as before for the interviews?

 The authors use the TRL concept, but it is not clear how the TRL was assigned to each product, by the authors? Directly by the manufacturers? A combination of both?

Results

 I would suggest implementing the graphical display of the results. It would give the reader immediate access to the information. For example, a graph with the percentages of the type of products and another with the distances of the feedstock from the processing would be appreciated.

 Again, a scheme of the main obstacles for introducing the products into the market would be appreciated.

 About CLT, a concern is the use of structural adhesives, which may increase the impact of the product and the difficulties at the end-of-life. This is mentioned further on about the lignin-based adhesives, but comments should be added here too.

 Could the authors add more info about the environmental impact of the processing and at the end-of-life of the glycols, as well as bioplastics?

 Could the authors mention some examples of wood-based composite?

Conclusions

 “decreasing the competition for land with food and feed production.” If the demand for bio-products and therefore biomass increases, it would likely lead to a competition for land with food and feed production. The authors state the opposite, so I think that they should explain better what they mean here.

Is the study design appropriate and does the work have academic merit?

Yes

Is the work clearly and accurately presented and does it cite the current literature?

Yes

If applicable, is the statistical analysis and its interpretation appropriate?

Not applicable

Are all the source data underlying the results available to ensure full reproducibility?

Yes

Are the conclusions drawn adequately supported by the results?

Yes

Are sufficient details of methods and analysis provided to allow replication by others?

Yes

Reviewer Expertise:

Chracterization of wood and wood-based products, mostly for strucutral uses.

I confirm that I have read this submission and believe that I have an appropriate level of expertise to confirm that it is of an acceptable scientific standard.

# Response

**Published:** 2022-06-10

**Authors:** Mariana Hassegawa

**Affiliations:** European Forest Institute, Finland

*The authors would like to thank the reviewer for the comments and time dedicate to review our manuscript. We have tried to respond as best as possible to the points raised by the reviewer. Our responses are shown in italics, and changes made to the text of the manuscript are presented in this document underlined.*

 The article titled “Innovative forest products in the circular economy” aims to give an overview of the more or less innovative bio-based products coming from the forests, which could help to tackle climate change. It is well written and organized, sound and logical in its methodology and exposition of results. It seems useful to have an overview of the actual level of development for the bio-based products coming from the processing of the forest raw resources. In some parts, it is a bit too general (wood-based components and biochemical), but it is most likely due to the high number of different products to be included in these categories. However, the literature is up-to-date and comprehensive, for further information. Therefore, most of my comments in the following aim to improve the readability of the work.

Introduction The sentence “Because of these concerns, the European Union (EU) has banned the production and consumption of single-use plastics (European Parliament and Council 2019), which would also have implications on bioplastics that are technically equivalent to fossil-based plastics (Brodin et al., 2017).” means that the same statement of the European Commission is valid both for fossil-based and bioplastic? The citation is not present in the list of references, by the way.

***Response from the authors:*** *Yes, if the bioplastic product is made with chemically modified polymers, whether natural or not, it is subjected to the same directive. We have added these clarifications to the text.* *Regarding the reference, it can be found in the reference list: Brodin M, Vallejos M, Opedal MT, et al.: Lignocellulosics as Sustainable Resources for Production of Bioplastics - a Review. Journal of Cleaner Production. 2017;162:646–64. 10.1016/j.jclepro.2017.05.209* *“Because of these concerns, the European Union (EU) has banned the production and consumption of single-use plastics (European Parliament and Council of the European Union, 2019). Since this EU directive applies to all plastics produced with chemically modified polymers, whether from natural or synthetic sources, it would also have implications on bioplastics.”*

 About the aims: “To which extent are these forest products compatible with existing value chains?”: as one of the demands of the research, it appears not always clearly stated along with the description of the several products.

***Response from the authors:*** *Thank you for pointing that out. Statements were added throughout the manuscript to clarify and emphasize to which extent the products were compatible to existing value chains. Below are the (underlined) sentences included in the manuscript to indicate to which extent the products were compatible to existing value chains.* *“ At first, the development and improvement of the technology to produce CLT was rather slow (Karacabeyli & Gagnon, 2019); however, in the early 2000s, its value chain was developed and the production increased with the interest for new sustainable building materials, product approvals and stronger marketing strategies (Karacabeyli & Gagnon, 2019). In the past few years, there has been an increase in interest for CLT due to the development of the wood construction sector.”* *…* *“ These new textile fibres will be part of the existing wood-based fibres value chain, placed together with viscose and lyocell.”* *…* *“Bio-based glycols can be produced from sugars extracted from agricultural crops, but also from woody biomass, in particular from broadleaved trees such as beech. Thus, glycols made from woody biomass are considered drop-in in the value chain for downstream applications. If demand for this type of feedstock increases, there is a future possibility to do breeding and clone selection aiming for trees with appropriate saccharification properties. Woody biomass would normally be locally sourced, and availability is not predicted to be a problem as there is a variety of possible sources.”* *…* *“However, the production of bioplastics from side streams of the wood industry is technically challenging and frequently economically unviable (Brodin et al., 2017). From the perspective of the forest sector, there is a need for a new value chain to produce these chemicals and bioplastics.”* *…* *“One of the lignin-based phenolic adhesives that can be found in literature had an estimated TRL of 8 (University of Bologna and Fraunhofer ISI, 2018). The development of lignin-based adhesives is mostly connected to the pulp and paper industry. Some adjustments will be needed to the value chain to include biorefineries that can fractionate the black liquor into value-added chemicals to produce lignin-based adhesives.”* *…* *“In addition, crude tall oil can be used to produce, among other chemicals, ethylene and the bioplastics produced from this chemical compound (De Bruycker et al., 2014). The production of bioplastics from tall oil would be connected to a certain extent to the pulp and paper industry. However, adjustments are needed to the value chain to include biorefineries that can fractionate the black liquor from the pulping process and produce bio-naphtha from crude tall oil.”* *…* *“Products that combine small wood elements, such as particles and chips, with a binding agent or thermoset polymer are called wood-based composites or wood-thermoplastic composites. These products have been used for many decades as construction material (in decking, siding, roofing among others), but have recently gained more applications due to new technologies and process improvement. The value chain for the new wood-based composites has already been developed, at times being adapted from traditional value chains.”* 

Methods: As concerns the interviews, 12 organizations were selected from a pool of 39 stakeholders. But the 10 interviews were few and the authors decided to go with an online survey. Why they did not try with the interview of the all 39 stakeholders? About the survey, again 39 stakeholders are cited. Are they the same as before for the interviews?

***Response from the authors:*** *We selected 12 organizations to be contacted for the interviews because it was used primarily for scoping of innovative products. We were able to interview 10 of the 12 organizations, and this success rate was adequate for the objective of the interviews and this stage of the study. We deemed it unnecessary to interview all 39 organizations as a large portion of the developments on wood-based products would likely be covered during the interviews with the 12 stakeholders or subsequently during the literature review.* *The survey was used to complement and validate the information gathered during the interviews and the literature review. The 39 organizations contacted for the survey belonged to the pool of stakeholders – the same group mentioned when describing the interviews. Clarifications were added to the manuscript.* *“Information on the development and characteristics of new or emerging products and technologies is not often publicly available. Therefore, we collected qualitative information and quantitative data using a combination of data collection methodologies, including interviews, a survey, and a scientific and grey literature review. Firstly, the semi-structured interviews would allow us to obtain more detailed answers from the participants (de Leeuw, 2008) and go deeper into discussions about products and aspects that were deemed more important to the organizations developing the products. Secondly, the online survey provided participants the flexibility to respond at their own time and pace, allow for anonymity, and reach a larger and more diverse group of participants (Braun et al. 2020). Thirdly and finally, the literature review allowed us to obtain specific information about products that were mentioned during the study. These multiple methods of data collection would help reduce the errors associated with using a single method (Patton, 1999), by allowing us to triangulate the data from these three sources (Tassinari et al., 2021 ), examine the data for consistencies and discrepancies (Mathison, 1988; Barnum, 2021), and increase the robustness of our results.”* *…* *“As the next step, we complemented the information collected during the interviews and literature review with an online survey. We developed the survey to collect qualitative and quantitative data on feedstock, transportation distance from feedstock source to mill, TRL and time-to-market, estimated production volume, information on substitution, among other information. The 39 organizations from the industry and research institutes that formed the pool of stakeholders in this study were contacted through email and asked to complete an electronic survey on SurveyMonkey.”*

 The authors use the TRL concept, but it is not clear how the TRL was assigned to each product, by the authors? Directly by the manufacturers? A combination of both?

***Response from the authors:*** *The estimated TRL was obtained directly with the organizations participating on the study and from the literature. This has now been clarified in the manuscript.* *“The interview questionnaire was built with the main objective to scope for innovative products being developed by the organization. We also aimed to collect qualitative data about the production process and the value chain, as well as to get insights about the current and future markets.* *The organizations also indicated, whenever possible, the time-to-market and TRL*.” … *“Once the products were selected, we conducted a review of scientific and grey literature, as well as websites of the manufacturing companies and research institutes.* *The information collected during the review included – but was not limited to – quality and quantity of feedstock, general description of production process, global demand and production quantities, TRL and time-to-market (for new products), and end-of-life options.*”  

Results I would suggest implementing the graphical display of the results. It would give the reader immediate access to the information. For example, a graph with the percentages of the type of products and another with the distances of the feedstock from the processing would be appreciated.

***Response from the authors:*** *We added Figure 3 on the distances from feedstock to production facilities.*

 Again, a scheme of the main obstacles for introducing the products into the market would be appreciated.

***Response from the authors:*** *We have included Figure 5 indicating the main obstacles and their importance to the survey respondents.*

 About CLT, a concern is the use of structural adhesives, which may increase the impact of the product and the difficulties at the end-of-life. This is mentioned further on about the lignin-based adhesives, but comments should be added here too.

***Response from the authors:*** *We have included a paragraph to indicate some of the potential environmental impacts related to the use of adhesives in engineered wood products and a mention to the development of bio-based adhesives.* *“ One of the issues that may arise with the use of CLT is related to the environmental performance of the adhesive used in this type of engineered wood product. The adhesive used in the production of CLT is usually formaldehyde-free polyurethane, but phenol-resorcinol formaldehyde and emulsion polymer isocyanate are other commonly used adhesives (Verkerk et al., 2022). In the past few years there has been an increased interest in developing bio-based options for adhesives to improve the environmental performance of products (i.e., reducing the carbon footprint, reducing toxicity, reducing energy input during production, and improving biodegradability) (Heinrich, 2019; Siddiqui, 2013).*” 

 Could the authors add more info about the environmental impact of the processing and at the end-of-life of the glycols, as well as bioplastics?

***Response from the authors:*** *Some information about environmental impacts and end-of-life options for bioplastics and glycols were added to their respective sub-sections.* *“Most MPG is derived from petroleum, but it can also be produced from plant-based glycerine or from glycerol that can be obtained as a side-product from the production of biodiesel. The environmental impact of bio-based glycerine varies depending on feedstock and production process. As for glycols, the environmental impacts can be attributed to a multitude of factors. The feedstock used for production should be renewable and transport of raw material limited to short distances. An environmental impact assessment comparing petroleum-based and bio-based glycols indicated a burden shifting towards the latter for indicators related to acidification potential, marine and terrestrial eutrophication potential and use of land and biotic resources (Nachtergaele et al., 2019). This study was based on glycols from agricultural crops and tallow, but hints to issues that may arise with the production of bio-based glycols. Because glycols produced by wood-based industries are still quite new, there is lack of information when it comes to their environmental impacts.”* *…* *“ One possible issue with bioplastics is related to the end-of-life options for the material. While using biomass as feedstock may help solve issues related to the dependance of non- renewable resources, it may not necessarily contribute to reducing plastic pollution, as bioplastics are not necessarily biodegradable, compostable or recyclable (Maier, 2018; Tenhunen and Pöhler, 2020). For this reason, it is important to take sustainability aspects into consideration during product conception and manufacture and have adequate disposal of the material at the end of its life to avoid pollution, especially caused by microplastics (Neves et al., 2020). It is also important to mention that currently many bioplastics are only partially bio-based. Bio-PET, for instance, is produced from ethylene glycol from biomass. However, it also requires terephthalic acid in the production process, which is only available commercially from fossil sources, resulting in a bio-PET that is approximately 30% bio-based (Spekreijse et al., 2019).”* *…* *“Some bioplastics from tall oil, such as the barrier films for liquid packaging, can be recycled with paperboard, which helps improve the circularity of these products. A constraint for the development of this segment would be the availability of the feedstock, as tall oil is already used for many bio-based products (De Bruycker et al., 2014). Disadvantages related to this material are related to the end-of-life options for the product, as previously mentioned for bioplastics from wood sugars.”*

 Could the authors mention some examples of wood-based composite?

***Response from the authors:*** *We had given already some examples of wood-based composite products (e.g., countertops, furniture, beverage straws) but clarified the original sentence and we added a couple more examples.* *“Plastic products can be substituted by durable wood-based composite products (e.g., countertops, furniture , containers) and disposable wood-based composite products (e.g., beverage straws , cotton swab sticks).”*  

Conclusions: “decreasing the competition for land with food and feed production.” If the demand for bioproducts and therefore biomass increases, it would likely lead to a competition for land with food and feed production. The authors state the opposite, so I think that they should explain better what they mean here.

***Response from the authors:*** *Our statement relates to the production of non-food and non-feed agricultural crops, such as cotton, and how they can compete for land with other crops. We revised the sentence to make this clearer.* *“Besides contributing to decreasing GHG emissions and storing carbon, the innovative forest products can also tackle other environmental issues typically associated with their non-wood counterparts, such as reducing pollution and waste generation, and decreasing the competition for land with food and feed production , as is the case for the production of cotton and other non-food agricultural crops.”*  

***Other changes to the manuscript*** *Besides minor changes to the manuscript, the results presenting the obstacles for introducing the products to the market were revised and updated:* *“Among the survey respondents who indicated the greatest obstacles for introducing their products to the market or increasing their market share, 29% mentioned the cost compared to fossil-based or GHG-intensive products as being the most important hindrance (Figure 5). Other difficulties were also mentioned as the most important aspect, with respondents mentioning as barriers the low feedstock availability, the production scale needed to disrupt the market, and the low availability of venture capital and governmental support. According to the respondents, the second greatest obstacle was related to the shift in production scale from pilot to full scale. Respondents considered technical difficulties an important to slightly important obstacle. Customer preference for traditional (i.e., fossil-based, or GHG-intensive) products was considered only a slightly important obstacle. Other difficulties mentioned by respondents were the high price of raw material, and the supply chain and market development. Among the survey participants, 38% indicated these difficulties could be largely alleviated by EU policies, 33% said that they could be slightly alleviated, and 29% did not know or did not answer the question.”*
